# Medical, ethical and personal dimensions of parent–offspring conflicts

**DOI:** 10.1093/emph/eou008

**Published:** 2014-04-08

**Authors:** Bernard Crespi

**Affiliations:** Department of Biological Sciences, Simon Fraser University, 8888 University Avenue, Burnaby, BC, Canada V5A 1S6

**Keywords:** evolutionary medicine, medical ethics, parent–offspring conflict

Among the most fundamental and intimate of human reproductive processes are fetal development and infant care. As first described by Haig [1], genetic conflicts centrally mediate such maternal–offspring interactions, and risks for a broad swath of major medical conditions, including pre-eclampsia, gestational diabetes and intrauterine growth restriction, appear to substantially involve dysregulation of evolved systems for conflict. In the current article, Haig [2] explains how infant suckling and sleep also represent central arenas for parent–offspring conflict, here over the length of inter-birth intervals. Post-natally, notable medical and public health risks, which also appear to be underlain in part by such conflicts, include failure to establish effective breast feeding or harmonious parent–infant interactions [3].

The profound implications of Haig’s insights into the roles of evolutionary conflicts in fetal, infant and maternal health are matched only by the remarkable absence of understanding, appreciation or application of such evolutionary principles among the research and clinical medical communities, or the general public. I explore this gap, and the relevance and practical applications of parent–offspring conflict for medicine, public health, ethical decision making and personal experience. My goal is thus to determine how the applied dimensions of parent–offspring conflict theory can be made more useful to increasing human health and well-being.

Our first dimension is medical. For doctors, ‘mother–offspring’ or ‘maternal–fetal’ conflict refers to situations where one or both parties suffer from a serious medical condition, but treatment to help one would impact badly on the other [4]. Severe pre-eclampsia represents a paradigmatic case, whereby sometimes the mother’s life may be saved only by dangerously premature delivery of the baby. For biologists, mother–offspring conflicts likewise involve tradeoffs between health of the two parties, which follow here from divergent, evolved phenotypic optima for fitness. Disorder, disease or reduced health ensue when conflict systems become dysregulated, when energy is squandered on conflict interactions or when one party more or less ‘wins’ to the detriment of the other [5, 6].

Primary limitations of conventional medical approaches in such conflict situations are (i) that symptoms (specific correlates) of disease may be considered as deleterious, and be suppressed by treatment, when they actually represent beneficial conditional defenses of one party [1] and (ii) that potential causes of disease may be overlooked. For example, Yuan *et al.* [7] and Haig [8] describe evidence that pre-eclampsia is mediated by fetal-placental release of one or more chemicals that damage maternal endothelium, with the subsequent higher maternal blood pressure (usually) benefitting fetal growth. Evolutionary perspectives yield novel hypotheses of causation, and their primary efficacy stems from directing data collection along new, promising paths. However, the general lack of permeation of such perspectives into the minds of doctors and medical researchers, over 20-plus years of opportunity, suggests that such syntheses of proximate with ultimate approaches will only happen by evolutionary biologists themselves establishing research links in medical communities, and by targeted teaching of medical-evolutionary thinking especially at the undergraduate and early-graduate levels. Evolutionary reasoning, and especially the logic and dynamics of evolutionary conflicts, do not naturally pervade the human psyche, or the conceptual frameworks of medical science.

Our second dimension is ethical. Haig [2] takes us to the edge of this minefield where evolution and medicine overlap with morality, law and religion, which is especially explosive in the context of human reproduction. To venture in, let us consider evolutionary biology simply as a guide to understanding the sources of morality [9, 10] and human reproductive behavior. On the one hand, morality and ethics center on fairness and justice for all, as expressed in systems of indirect reciprocity, and as conceptualized by individuals according to common societal interests in relation to their own. On the other hand, striving to maximize inclusive fitness should centrally engender control over one’s reproductive and parental decision making even to the possible detriment of others: personal reproductive and parenting liberty, as it were. With regard to mother–offspring conflict, mothers are expected to strive to further their own inclusive fitness interests (usually, under a veil of ignorance that they do so), in the contexts of interactions with offspring, spouses, other family members and the moral prescriptions of society at large—all of whose interests may be more or less divergent from theirs.

What can evolutionary biology offer here, to further human well-being most generally? I suggest that recognition and characterization of evolutionary conflicts and tradeoffs represent a key first step toward reducing and alleviating them, through societal and public-health policy and interventions, and education. For example, mother–offspring conflicts, and tradeoffs, regarding inter-birth intervals have already been reduced, in developed countries, by enhanced nutrition and infectious-disease controls. In situations of unresolvable conflict, ‘fairness’ to both parties would appear to involve an intermediate phenotypic optimum with regard to effects on inclusive fitness—to the extent that such an outcome can be achieved or attempted. Knowledge from evolutionary biology need not rationalize or motivate ethics, but it can help us to reach the ethical system that has been deemed most suitable or appropriate. Most importantly, ethics is *about* conflicts of interest [9], so such conflicts should be understood, in evolutionary as well as other frameworks, to develop moral systems that are meaningful, practical and enhance human welfare.

Our third dimension is personal: how can understanding of parent–offspring conflicts be useful in our own lives, with our own families? As noted by Haig [2], we predict in parents a certain ambivalence toward offspring: a mixture of love and infuriation for a crying, night-waking infant, for example, coupled with shame or repression of thoughts that do not reflect unconditional altruism. Recognizing that children are expected to solicit more energy and time than we might choose to provide them should temper feelings of parental or offspring inadequacy or intractability; likewise, expectations of sibling rivalry can motivate conscious strategies to pre-empt or reduce it, to the benefit of all concerned. Among individuals and families, such ambivalence, and mixtures of altruism and conflict, may also underlie risk for specific maladaptive extremes of parental and offspring behavior, exemplified for example in colic, insecure or overly secure attachment, or parental neglect, abuse or over-involvement [3, 11]. Given the lifelong health impacts of fetal and child physical and psychological development, we owe nothing less to our future generations, nor they to us, than to better understand the interfaces of cooperation with conflict in this their most intimate of settings.
